# Beyond the Disease: Contextualized Implications of the COVID-19 Pandemic for Children and Young People Living in Eastern and Southern Africa

**DOI:** 10.3389/fpubh.2020.00504

**Published:** 2020-10-19

**Authors:** Kaymarlin Govender, Richard Gregory Cowden, Patrick Nyamaruze, Russell Murray Armstrong, Luann Hatane

**Affiliations:** ^1^Health Economics and HIV/AIDS Research Division, University of KwaZulu-Natal, Durban, South Africa; ^2^Human Flourishing Program, Institute for Quantitative Social Science, Harvard University, Cambridge, MA, United States; ^3^Paediatric-Adolescent Treatment Africa, Cape Town, South Africa

**Keywords:** COVID-19, Eastern and Southern Africa, health, well-being, children, young people

## Abstract

The coronavirus disease 2019 (COVID-19) pandemic has created extraordinary challenges and prompted remarkable social changes around the world. The effects of COVID-19 and the public health control measures that have been implemented to mitigate its impact are likely to be accompanied by a unique set of consequences for specific subpopulations living in low-income countries that have fragile health systems and pervasive social-structural vulnerabilities. This paper discusses the implications of COVID-19 and related public health interventions for children and young people living in Eastern and Southern Africa. Actionable prevention, care, and health promotion initiatives are proposed to attenuate the negative effects of the pandemic and government-enforced movement restrictions on children and young people.

## Introduction

The outbreak of severe acute respiratory syndrome coronavirus 2 (SARS-CoV-2) is likely to create unprecedented challenges in Eastern and Southern Africa (ESA), a region where health systems are fragile, socioeconomic inequalities exist, and public health crises of HIV and tuberculosis are rampant. Many countries in this region instituted nationwide public health control measures (e.g., social distancing requirements, stay-at-home orders) to minimize the spread of SARS-CoV-2 and reduce the burden of the coronavirus disease 2019 (COVID-19) pandemic on health systems. Although such measures are designed to flatten the curve of SARS-CoV-2 transmission, they often present unique direct and indirect consequences for specific subpopulations. This paper provides an analysis of the implications of COVID-19 and related public health interventions for the well-being of children and young people (CYP)^1^ living in ESA. We discuss responses that should be implemented to mitigate the negative effects of the COVID-19 pandemic on CYP in the region (for a summary, see Table 1).

**Table 1:** Summary of COVID-19 vulnerabilities and strategies for promoting well-being among children and young people in Eastern and Southern Africa.

**COVID-19 related vulnerabilities**	**Levels at which prevention, care, and health promotion interventions are targeted**			
	**Individual behavioral strategies (prevention, self-monitoring and early detection)**	**School-based and community level strategies**	**Health systems level strategies**	**Legislation/policy strategies**
**Existing health issues**				
•Unsuppressed viral loads and low CD4 counts •Undiagnosed HIV and TB	•Improve self-efficacy to adopt COVID-19 prevention behaviors (e.g., restriction of movement measures, hand washing, social distancing) •Improve self-efficacy for HIV viral load testing, TB testing, and SARS-CoV-2 testing •Self-identify COVID-19 symptoms and self-quarantine •Use self-support tools to promote medication adherence •Improve ability to monitor healthy levels of nutrition •Complete prescription refills early and obtain multi-month stocks of required medication	•Support integrated community prevention, surveillance, and early detection of HIV/TB and SARS-CoV-2 by linking CYP to community social and health services •Increase family- and community-based support for YPLHIV through alternative avenues (e.g., social media groups, online ART adherence clubs and peer support) that comply with social distancing requirements •Telephonic counseling for those with high viral loads •Capacitate YPLHIV to fulfill curriculum-based learning away from school (e.g., online platforms, community library, home schooling) •Monitor stigma, discrimination, and human rights abuses of YPLHIV during lockdowns	•Provide uninterrupted primary health care with intensified SARS-CoV-2 surveillance •Triage screening for SARS-CoV-2 and COVID-19 symptoms •Continue to allocate multi-month HIV and TB prescription refills, while monitoring for stock-outs •SARS-CoV-2 testing to include assessment of medication availability and adherence to medication among YPLHIV •Ensure pediatric high care facilities are available for severe COVID-19 patients •Monitor discharged and recovered COVID-19 patients	•Increase budgets to ensure HIV, TB, and SARS-CoV-2 testing and treatment programs are stepped up and continued during lockdowns •SARS-CoV-2 surveillance should be integrated into existing HIV/TB programs •Increase legislative capacity to respond to human rights abuses against YPLHIV during lockdowns
**Consequences of imposed restrictions and lockdowns**				
Health complications of physical inactivity and restricted mobility	•Improve self-efficacy for engaging in appropriate physical activity within the confinements of regulated spaces •Improve self-efficacy for maintaining a nutritional diet	•Caregivers and communities to support children with developing routines that incorporate physical activity and regular meals •Communities to support health and exercise activities when restrictions are eased •School-based information to emphasize and support weekly routines	•Surveillance systems to monitor food shortages and health and nutrition needs of CYP	•Accommodate physical activity needs of CYP into stay-at-home orders or homebound policies, particularly for those without access to special equipment or who have limited space in the home to exercise
Psychological distress precipitated by confinement and fear of contracting SARS-CoV-2	•Self-monitor psychological distress and seek appropriate support and services •Identify and utilize coping resources (e.g., friends, family, community leaders) •Maintain a routine and prioritize self-care	•Support community outreach health workers with detecting mental health issues and delivering basic mental health services to families •Families and communities to provide CYP with access to health workers for psychosocial support •School-based information to emphasize detection and treatment of mental illness	•Where possible, use innovative ways to provide mental health support to people during lockdown periods (e.g., telemedicine, web-based counseling and wellness services, SMS, information through cross-platform messaging and voice-over internet protocols [e.g., WhatsApp])	•Mental health services should be classified as an essential primary health service •Public health interventions must be child-friendly and sensitive to the capacities and vulnerabilities of children
Immunization programming disruptions	•Caregivers to ensure that children are vaccinated in accordance with recommended immunization schedules	•Community outreach/door-to-door immunization for children during lockdowns	•Universal immunization should be integrated into the COVID-19 response and considered an essential service	•Accelerate research on the development of a safe and effective SARS-CoV-2 vaccine
	•Caregivers to ensure neonatal BCG vaccination to be given to all infants in high TB burden settings •Caregivers to ensure infants who have not yet received immunization require a catch-up vaccination at the nearest health facility	•Provide community-wide education on vaccination guidelines and immunization schedules •School-based immunization campaigns to reach older children and adolescents, even when schools are closed		•Ensure equitable access to COVID-19 vaccine once available
Learning during school closures	•Improve basic skills needed to access online and distance learning modalities •Make use of available educational material delivered through various multimedia services (e.g., radio, television, local community center where social distancing in place)	•Schools to consider ways of making learning materials easily accessible to students •Launch multi-media learner support initiatives (e.g., radio and television educational programming) •Where possible, teachers to be trained on conducting remote-based learning and encouraged to stay in contact with learners (e.g., radio, television) •Caregivers to assist with essential home-based learning skills (e.g., reading, writing, mathematics) •Communities to support remote learning through exchange of information on where materials can be accessed •Create safe spaces (e.g., community centers where social distancing is in place) to collect educational material	NA	•Implement a national policy to mitigate the immediate impact of school closures and facilitate continuity of education for all students through remote learning •Provide household grants to support out-of-pocket costs to access learning material
Re-opening of schools	•Improve education on prevention, detection, management, and treatment of COVID-19	•Support schools to institute and adhere to COVID-19 safety and risk mitigation measures	•Capacitate local health systems to be responsive to COVID-19 outbreaks in schools	•Decisions on re-opening of schools should consider context-specific issues in combination: SARS-CoV-2 epidemic trajectory among CYP, SARS-CoV-2 transmission rates, prevalence of severity of COVID-19 related illness and mortality rates among CYP, ongoing negative impacts on immunization programs, school feeding programs and other public health programs, capacity of schools to adhere to COVID-19 risk mitigation and safety measures, and evaluation of the benefits of classroom-based instruction vis-à-vis remote learning
Human rights violations	•Improve self-efficacy to access to helplines and self-help empowerment information •Encourage members of young key populations to keep in “social contact groups” to monitor victimization and abuse and have knowledge on how to access support services	•Communities and local NGOs to ramp up information campaigns on monitoring and reporting of human rights violations during lockdowns •Community and NGO services to ramp up messaging campaigns on access to safe spaces, shelters, and support groups •Deliver family-integrated protective behaviors education to children and caregivers through television and radio •Advocacy groups to educate sex workers and other vulnerable groups of their human rights and where to report violations of human rights •Provide helpline contact details for reporting child abuse and seeking support	•During lockdowns, child protection services and workers must be designated as essential services and resourced to access and support children with age-appropriate services, safe e-education platforms, and cost-free child helplines for children to report the occurrence of abuse or violence •Integrate human rights programming into the COVID-19 health systems response •Strengthen case management and multi-sectoral referrals to holistically support at-risk and vulnerable children	•Parliamentary process and civil society organizations to serve as “watch dogs” to monitor law enforcement misconduct and helping to ensure offenders are held accountable for acts of discrimination and violence •Provide extra legislative capacity to communities that need assistance dealing with human rights violations (e.g., victims discrimination, xenophobia, violence, imprisonment) during lockdowns •Shelters and psychosocial support services for marginalized populations must be considered an essential service in every country
Financial and food insecurity of households	•Caregivers of households to monitor and report food shortages and lack of funds to purchase essentials (e.g., medicine, hygienic products)	•Communities and local NGOs need to support impoverished families with accessing social relief funds and food distribution programs •Impoverished families to be linked with appropriate social and development services, with NGOs and communities monitoring for bottlenecks and breakdowns in service access and delivery	•Health workers to identify children at risk of hunger/malnutrition and support them through rapid linkage to community food distribution and feeding programs	•Increase government spending on social grants to improve household food security •Roll-out large-scale food assistance programs (e.g., food banks) in both urban and rural areas •Keep food supply chains open and accessible to communities during lockdowns •Parliamentary process and civil society organizations to serve as “watch dogs” to monitor corruption in distribution of resources

*BCG, Bacille Calmette-Guérin; COVID-19, coronavirus disease 2019; CYP, children and young people; NGO, non-governmental organization; SARS-CoV-2, severe acute respiratory syndrome coronavirus 2; SMS, short message service; TB, tuberculosis; YPLHIV, young people living with HIV*.

## Medical Care Needs of Children Living With Unsuppressed Viral Loads, Low CD4 Counts, and Tuberculosis Infections

Previous disease outbreaks have demonstrated that when health systems are overwhelmed, deaths from vaccine-preventable (e.g., tuberculosis) and treatable conditions (e.g., HIV) tend to increase. COVID-19 is likely to adversely affect the many CYP living with HIV in the region, especially those who are not aware that they are HIV positive and those with unsuppressed viral loads and low CD4 counts. Estimates from countries in ESA (e.g., Kenya, South Africa) indicate that up to 37% of HIV positive CYP are living with unsuppressed viral loads (3, 4). Unsuppressed viral loads (and low CD4 counts) increase vulnerability to opportunistic infections, including respiratory-related conditions (5). HIV testing must be paired with SARS-CoV-2 testing to detect the concurrent presence of these viruses in CYP. Those who test positive for SARS-CoV-2 should be monitored closely (if asymptomatic) or treated for COVID-19 symptoms, whereas those who test positive for HIV should immediately be placed on antiretroviral treatment (ART). The latter is particularly important for young people who are at increased risk of being severely affected by COVID-19, including those who have not disclosed their HIV status and those who defer seeking HIV treatment during the pandemic. For CYP on ART, continuation of comprehensive ART is crucial to achieving optimal adherence and viral suppression.

Recently modeled projections indicate that a 6 months disruption of ART could lead to an additional 465,000 AIDS-related deaths in ESA in the next 12 months (6). As countries in the region implement COVID-19-related public health control measures, there is a need to allocate multi-month prescriptions and refills to reduce the frequency of visits to clinical settings and maintain access to HIV prevention services, including condoms and pre-exposure prophylaxis (7). This will ensure that patients have enough treatment during stay-at-home orders and limit unnecessary visits to health care facilities, thereby reducing the risk of exposure to SARS-CoV-2. Children with unsuppressed viral loads who contract SARS-CoV-2 will need to be placed immediately in high care facilities to manage complications from co-infections.

## Disruptions to Immunization Programs

Long-term stay-at-home orders that have been implemented to contain the spread of SARS-CoV-2 have disrupted vaccination campaigns and immunization activities, which increases the risk of children contracting other infectious diseases. Measles immunization campaigns have been delayed in 24 countries and will be canceled in 13 others, with millions of children missing out on immunization activities during the pandemic (8). Many countries in ESA already had sub-optimal rates of immunization for vaccinable diseases (e.g., measles, polio) before the COVID-19 pandemic. For example, 2018 estimates indicate that Angola and Ethiopia accounted for 45% of all infants in ESA who were un- or under-vaccinated for diphtheria, tetanus, and pertussis (9). Immunization activities in this region are likely to be disrupted by social responses to COVID-19 (e.g., reluctance to attend vaccination sessions for fear of exposure) and the effects of public health control measures (e.g., border closures and travel disruptions can impact vaccine accessibility). These conditions raise the risk of sudden outbreaks of vaccine-preventable diseases occurring when social distancing restrictions are eased. For children who already have a compromised immune system (e.g., those living with HIV), likelihood of mortality from vaccine-preventable conditions (e.g., measles) is higher if immunizations are not received (10).

While acknowledging the importance of initiating measures to minimize the spread of SARS-CoV-2, delivery of immunization services is essential to maintaining the health of children through vaccinations for preventable diseases. Particularly in ESA where health care systems are under-resourced, finding a balance between containing transmission of SARS-CoV-2 and continuing immunization programs is critical. Planning is needed to ensure that unvaccinated children are prioritized by immunization initiatives (e.g., large-scale, home-based immunization campaigns) and developing contingency plans to circumvent immunization campaign disruptions caused by homebound orders related to COVID-19.

## Physical, Psychological, and Social Consequences of Human Mobility Restrictions

As SARS-CoV-2 rapidly spreads across the world, it is inducing a considerable degree of fear and anxiety among people. Measures that have been implemented to contain the virus, including restrictions on freedom of mobility, limits to physical social contact, and imposed isolation and quarantine, can negatively impact the health and well-being of CYP (11). The consequences of stay-at-home orders are likely to be exacerbated in low-resource countries where financial capacity to support CYP is limited (12).

Stress that is triggered by homebound orders can weaken immune systems of growing children and increase their susceptibility to infections (13). CYP who are forced into sedentary lifestyles are at higher risk of developing non-communicable chronic illnesses (e.g., diabetes, hypertension), which is already a growing concern in low-resource contexts such as ESA (14). Because young people living with HIV are more vulnerable to mental illness, especially depression (15), coping with a public health emergency like COVID-19 might compound pre-existing psychological distress.

Restrictions to mobility imposed by lockdowns will make it difficult for CYP living in ESA to access health services. As funding and health care services are scaled up to manage COVID-19 and its psychosocial effects, it is important that essential counseling and social support services remain accessible. Innovative approaches need to be developed and implemented to provide mental health support to CYP during the COVID-19 pandemic (e.g., telemedicine, virtual peer support, online counseling and wellness services). Where such services are not feasible, community health workers and families need to be supported to care for CYP. Relaxing lockdown restrictions by creating opportunities for CYP to engage in physical activity will improve physical and psychological health.

In ESA, unemployment rates remain relatively high, with young people being disproportionately affected (16). Most of the employable young people in the region rely on the informal sector for income. In countries that were confronted with food insecurity before the pandemic (e.g., Zimbabwe), stringent public health control measures that are now linked to COVID-19 are exacerbating hunger and poverty among young people. It is crucial that governments institute social protection measures to cushion the informal economy and provide food subsidies for those living in poverty.

## Impact of School Closures on Health, Safety, and Learning

As a result of COVID-19 stay-at-home orders, many children have experienced a disruption in formal education. Nationwide school closures are likely to have negative implications for the educational experiences of many children, especially those living in ESA where schools lack sufficient infrastructure to support the educational needs of children while stay-at-home orders are enforced. Government-sponsored school nutritional programs (e.g., feeding schemes) are prevalent in many countries in the region (17). Closing schools immediately restricts access to these programs, which many children depend on. Poor nutrition has been associated with worse educational outcomes in children, weakened immune systems, susceptibility to opportunistic infections, and premature mortality (18). During periods of prolonged school closures, there is a need to ensure children continue to have access to food. South Africa recently increased household funding through a child support grant that provides an additional R300 per child and R500 per caregiver each month (19). Similar initiatives are required in other countries in the ESA region.

School closures during times of crisis can heighten children's risk of exploitation, abuse, and violence (20). During homebound restrictions, families and communities need to be vigilant and protect children from harm. Countries may benefit from adopting the seven strategies outlined in the INSPIRE package (21). INSPIRE is designed to assist countries and communities to focus on prevention programs and services that have the greatest potential to reduce violence against children. This package has been successfully used in low- and middle-income countries, including those in ESA. During stay-at-home periods, social and child protection services must be designated as essential services and sufficiently resourced to support children with age-appropriate services, safe e-education platforms, and cost-free child helplines for children to report incidences of abuse or violence. Caregivers also need to be offered guidance on communicating in clear and sensitive language to children about risks, concerns, and protective measures related to SARS-CoV-2 transmission and infection.

Children from many impoverished households in ESA are also likely to fare poorly at homeschooling or distance learning due to challenges accessing electricity, electronic devices (e.g., computers), and the internet. Government and private sector partnerships with schools are needed to provide learners and caregivers with resources to facilitate meaningful remote learning opportunities. Basic Education Ministries should identify ways of supplying learners with printed reading materials through community health workers and community centers that are applying COVID-19 safety measures. Caregivers of children must be given support to implement simple routines that maintain typical eating windows, incorporate time for educational activities (e.g., reading), and include recreational activities that adhere to public health control measures. Teachers should be encouraged to stay in regular contact with learners and caregivers during school closures to ensure that learners understand and can engage with educational material. Teachers also need to be trained to remotely teach children living with disabilities, and caregivers should be given assistance with making distance learning accessible to children with disabilities. In low-income countries, radio and television education broadcasts may be more effective than e-learning (22). Rapidly creating age- and grade-appropriate educational radio and television programs in different languages can support learning during school closures.

There is evidence to suggest that most children who are infected with SARS-CoV-2 have mild symptoms or are low transmitters of the virus (23). As countries consider whether or not to re-open schools, the best interests of children and overall public health should be considered. Decisions need to be based on localized prevalence rates of SARS-CoV-2, the ability of schools to adhere to COVID-19 safety regulations, and an assessment of the benefits of classroom-based instruction vis-à-vis remote learning (24).

## Disproportionate Implications of Violence, Human Rights Abuses, and Limits on Access to Services for Marginalized Groups

The COVID-19 pandemic is accentuating social-structural inequalities, which tend to disproportionately affect marginalized people and those living in financially precarious situations (25). As countries implement public health policies to minimize transmission of SARS-CoV-2, young girls and women, people who identify as LGBTI, people who engage in sex work, informal traders, and street children are more likely to be targets of police brutality (26, 27). There have also been reports of misuse of emergency powers by governments to target marginalized and vulnerable populations (27). For example, a number of LGBTI shelter residents in Uganda were falsely arrested and incarcerated for approximately 50 days on the pretext of violating COVID-19 lockdown regulations (28). Young sex workers may have fewer avenues to protect themselves and are more prone to being victims of violence from police and other sex workers (29). Some younger sex workers may have children, which might increase their risk-taking propensity as they search for income and food to support their families.

Access to contraceptives is also a challenge with the imposition of COVID-19 stay-at-home requirements. Restricted mobility, reduced availability of public transportation, and closures of non-essential retail outlets and youth centers limits the capacity of young people to access contraceptives. Shortages of these commodities may precipitate risky sexual practices and unintended pregnancies (30), both of which were already long-standing issues in ESA before lockdowns were imposed in response to COVID-19 (31, 32). For young people living with HIV, condom shortages may increase the likelihood of onward HIV transmission. Further, fear associated with contracting SARS-CoV-2 is preventing individuals from attending public clinics (33). Closure of non-governmental organizations (NGOs) and community centers also places additional strain on the homeless and street children who ordinarily rely on those sources for food, clothing, and basic hygiene products.

As COVID-19 stay-at-home orders confine people to their homes, some young women may not have the opportunity to distance themselves from perpetrators of abuse or seek in-person support and health services for experienced abuse. Countries in ESA (e.g., Kenya, South Africa) have reported increases in the incidence of gender-based violence since COVID-19 homebound orders began (34, 35). Periods of confinement or lockdown accentuate the need to reach the most vulnerable groups with social safety nets. While it may be difficult to reach vulnerable populations when country-level COVID-19 public health control measures are in place, civil society organizations and NGOs need to actively monitor incidents of human rights violations by law enforcement and military personnel who enforce stay-at-home orders and social distancing measures. More broadly, civil society organizations ought to be involved in mitigating unintended consequences of the pandemic, including gender-based violence, discrimination, and food insecurity.

NGOs with established networks are more likely to have access to marginalized populations and should act as conduits between recipients and donors that offer shelter, access to food, and other essential services. Retail shops and youth centers that provide sexual and reproductive health services should be classified as essential services to ensure continued provision of contraceptives and medical treatment to young people. Upholding the rights of all citizens, including marginalized groups, should be a cornerstone of the COVID-19 response. Legal and psychosocial support services should also be accessible to CYP who may require “crisis response” interventions.

COVID-19 has provoked social stigma and discriminatory behaviors (36). People who are already living with a stigmatized health condition (e.g., HIV) could face dual stigmas if they contract SARS-CoV-2. Stigmatization can deter health-seeking behaviors and contribute to more severe health problems (37). Local broadcasters ought to regularly feature medical experts and health scientists to support the dissemination of accurate information about individual and group vulnerability to COVID-19, safe prevention and health promotion measures, and effective treatment approaches. With so many sources of information available to CYP, government-supported initiatives are needed to ensure that the public is informed about where to acquire credible information about COVID-19. Caregivers must be empowered to provide accurate, age-appropriate information to children about stigma and supervise exposure of CYP to information about COVID-19. Innovative, ongoing support services are also needed to assist CYP who are infected with SARS-CoV-2 or recovering from COVID-19 to cope with stigma and its psychosocial consequences.

## Food Insecurity in Families and Communities

In ESA countries that have been affected by COVID-19, public health measures designed to control the spread of SARS-CoV-2 has stalled economies and severely impacted the livelihood of people. Many people in ESA are employed informally, have low-paid contract positions, or receive hourly wages. The abrupt closure of many businesses (formal and informal) has resulted in a sudden loss of income for numerous people, with household food and health security being threatened. The World Food Programme (38) has warned that more than 200 million people could be pushed into acute food insecurity by the COVID-19 pandemic, many of which will be residents of ESA countries. ESA also has an immense number of children orphaned by AIDS Double orphans, in particular, are likely to end up living on the street, in youth- and child-headed households, or with extended family members who are likely to experience further financial strain because of the increased dependency ratio (39).

Food insecurity will limit the availability of nutritional food choices, which could detract from optimal immune system functioning and reduce the effectiveness of ART among those who are living with HIV. Addressing the impact of income loss in lower-income households through allocation of cash transfers can ease the burden of food insecurity. For example, South Africa has implemented the COVID-19 Social Relief of Distress grant that is paid to individuals who are currently unemployed and do not receive any other form of social grant. While cash transfers can assist many low-income households, this may not be sufficient to avert food insecurity. Large-scale roll out of food assistance programs (e.g., food banks) in both urban and rural areas is needed to supplement cash transfers, ensure access to life-sustaining food, and prevent social unrest and hunger riots. Food security at home could be improved through home-delivered meals facilitated by local organizations (e.g., NGOs, municipalities). School feeding programs need to be reintroduced, with the option of community sites becoming key distribution points that are accompanied by screening for COVID-19 symptoms, follow-up, and monitoring of children from affected households. Therapeutic nutrition ought to be provided to children who are malnourished or receiving ART. In the long-term, providing lower-income households with direct livelihood support through financed projects to develop small scale livestock and agricultural activities will both support child nutrition and mitigate the strain of food shortages and increases in food prices.

## Conclusion

The COVID-19 pandemic has prompted extraordinary measures around the world to slow the pace of SARS-CoV-2 transmission and minimize the public health consequences of the disease. Though necessary, some of these measures may have direct and indirect implications for specific subpopulations. Public health control orders should be cognizant of the unique needs of CYP, particularly those with underlying health conditions and who live in impoverished conditions. Countries in ESA will need to balance responding directly to the COVID-19 pandemic with upholding human rights and supporting CYP, particularly more vulnerable groups (e.g., children living with HIV, young women), to ensure food, education, and counseling services are available during government-enforced movement restrictions. More generally, the COVID-19 public health crisis highlights the importance of providing fiscal support to improve health systems and other institutional capacities in ESA, such as education and national security.

## Data Availability Statement

The original contributions presented in the study are included in the article/supplementary material, further inquiries can be directed to the corresponding author.

## Author Contributions

KG, RC, and PN conceptualized the manuscript. RC and PN led the writing of the manuscript. KG, RC, PN, RA, and LH provided critical revisions, edited, and finalized the manuscript.

## Conflict of Interest

The authors declare that the research was conducted in the absence of any commercial or financial relationships that could be construed as a potential conflict of interest.

## Footnotes

^1^*Children and young people* is an inclusive term that refers to any person aged 24 years or younger (1, 2).
